# Special Issue “Machine Learning Methods for Biomedical Data Analysis”

**DOI:** 10.3390/s23239377

**Published:** 2023-11-24

**Authors:** Cesar F. Caiafa, Zhe Sun, Toshihisa Tanaka, Pere Marti-Puig, Jordi Solé-Casals

**Affiliations:** 1 Instituto Argentino de Radioastronomía—CCT La Plata, CONICET/CIC-PBA/UNLP, V. Elisa 1894, Argentina; 2 Computational Engineering Applications Unit, Head Office for Information Systems and Cybersecurity, RIKEN, Wako-Shi 351-0198, Japan; zhe.sun.vk@riken.jp; 3 Department of Electrical and Electronic Engineering, Tokyo University of Agriculture and Technology, Tokyo 184-8588, Japan; tanakat@cc.tuat.ac.jp; 4 Data and Signal Processing Research Group, University of Vic-Central University of Catalonia, 08500 Vic, Catalonia, Spain; pere.marti@uvic.cat

## 1. Introduction

Machine learning is an effective method for developing automatic algorithms for analysing sophisticated biomedical data [1]. Due to the rapid development of artificial intelligence, the application of machine learning to biomedicine has shown promising results in recent years [2,3,4]. Machine learning is currently one of the most popular methods used in several biomedical areas, such as medical decision making [5], radiological image segmentation [6], track prediction for medical imaging [7], and others.

Despite rapid advances in the field of biomedicine-oriented machine learning, there is still a need for continuous development in a wide range of scenarios. For example, in the case of very diverse biomedical data that may correspond to different data types and structures, strategies such as data pre-processing, feature extraction, feature selection, data parameterisation, model selection, and training tricks will greatly affect the final performance of the algorithms employed and are often well designed [8]. In addition, special structures such as interpretable outputs, data augmentation, and unsupervised loss are popular in certain cases and may also vary due to different scenarios. 

The aim of this Special Issue was to collect novel contributions on machine learning methods for biomedical data to provide advanced insights and clear examples of their application in real biomedical scenarios. This Special Issue attracted the attention of researchers from all over the world. As shown in Table 1, a total of sixteen papers were submitted, ten of which were accepted after appropriate review. We were pleasantly surprised by the diversity of the contributors’ nationalities and the variety of applied science problems addressed, ranging from medical and health applications to specific industrial case studies. The authors of the published articles hail from 11 countries (USA, Poland, Finland, Lithuania, Netherlands, France, Pakistan, China, Serbia, the UK, and Spain) located in Europe, America, and Asia. The accepted list includes organisations from academia, industry, and laboratories.

The following sections summarise the accepted papers and their most relevant contributions, which are grouped into the following categories: biomedical diagnostics, biomedical care tools, health monitoring, industrial applications, and others.

## 2. Biomedical Diagnosis

Biomedical diagnosis is one of the most important applications of machine learning in biomedicine. Diseases such as heart disease, kidney disease, breast cancer, diabetes, Parkinson’s disease, Alzheimer’s disease, COVID-19, etc., have been thoroughly investigated regarding diagnosis with machine learning methods in previous work [9]. Interestingly, most of the contributions are related to specific applications in biomedicine. Four papers addressed different neuroscience problems or diseases. For example, in Contribution 1, Chauhan et al. (USA) presents an approach to diagnosing various stages of diabetes mellitus (DM) progression in the lower extremities. They collected dynamic pressure distribution data using pressure measurement templates and trained their model to diagnose whether a patient had prediabetes (PD), diabetes without peripheral neuropathy (D), or diabetes with peripheral neuropathy (DN). In Contribution 2, Far et al. (hailing from Poland, Finland, and Lithuania) proposed a method for reliably predicting preterm delivery using electrohysterogram (EHG) signals based on different weeks of pregnancy. They applied intrinsic mode functions (IMFs) obtained from empirical mode decomposition (EMD) for feature extraction and achieved competitive performance in the PE-TE and PL-TL groups compared with that achieved using state-of-the-art methods, wherein data were recorded before and after 26 weeks of gestation, respectively. In Contribution 3, Vajs et al. (Serbia) focused on the detection of dyslexic tendencies among Serbian children based on eye-tracking measurements. Children’s eye-tracking trajectories were recorded while reading for subsequent training in dyslexia recognition. In addition to nine conventional features, they also proposed five additional features (active reading time, the fixation intersection coefficient, saccade variability, fixation intersection variability, and the fixation fractal dimension) for this task. Their statistical analysis showed that the influence of colour has a high inter-subject variability. In Contribution 4, Marti-Puig et al. (Spain) developed a machine learning system to detect Ecological Momentary Screening (NSSI) using Ecological Momentary Assessment (EMA) data collected using the Sinjur application. These authors focused solely on emotions and developed a tree model to reveal which emotions allowed the system to determine whether new data belonged to an NSSI or non-NSSI class.

## 3. Biomedical Assistance Tools

In addition to diagnosis, problems regarding how to carry out medical treatment, patient care, and so on are equally crucial in biomedicine [10]. Machine learning methods can also function as tools to assist in addressing these matters. In this Special Issue, two papers describe approaches in which a machine learning method is used as a tool to help improve the treatment process. In Contribution 5, Khan et al. (hailing from France and Pakistan) proposed a multi-task learning (MTL) framework to predict gait trajectories. Kinematic data were recorded before and after botulinum toxin type A (BTX-A) treatment, and the Bi-Directional Long Short-Term Memory (Bi-LSTM) model was applied to map the gait sequences before and after treatment. The mapping results can guide the spasticity treatment process. In Contribution 6, Xie et al. (China) proposed a Multivariate Hybrid Attention Model (MVHA) to predict the imminent need for intravenous injection via jointly mining multiple time series. They designed a hybrid two-level attention mechanism model to capture the pattern of fluctuations and trends of different time series, including blood pressure (BP), peripheral arterial oxygen saturation (SpO_2_), heart rate (HR), pulse, and respiratory rate (RESP). They applied MVHA to the prediction of the imminent need for intravenous injections of critically ill patients in intensive care units (ICUs) and achieved promising performance compared to reference methods.

## 4. Health Monitoring

Machine learning methods are promising in health monitoring for disease prevention, especially with the great technological advances in wearable devices in recent years [11]. Accordingly, one of this Special Issue’s contributions proposes a health-monitoring algorithm deployed using a machine learning method. In Contribution 7, Shahbakhti et al. (hailing from Netherlands and Lithuania) aimed to use functional near-infrared spectroscopy (fNIRS) for respiratory rate (RR) estimation. They extracted five respiratory modulations and applied the fast Fourier transform for feature extraction and proposed a mean-based fusion method for RR estimation. Analysis of the individual modulations showed that the proposed fusion strategy outperformed the RR estimates. Their efforts overcome the limitations of RR estimation during physical activities.

## 5. Industrial Applications

This Special Issue also includes an article on the application of machine learning to practical industry-specific problems. In Contribution 8, Lopez-de-Ipina et al. (UK and Spain) addressed the problem of the risk management of workers with health disorders in the workplace by designing an intelligent risk management system for workers suffering from diseases. They presented a novel intelligent risk management system called HUMANISE. Monitoring video sequences were recorded, and an algorithm based on machine learning, intelligent agents, and computer vision was applied for risk management. Their experiments in a two-armed Cobot scenario showed the promise of their real-time support tool for coordinating and monitoring safe industrial environments.

## 6. Other Applications

Two very important machine learning problems, namely, (a) synergies between kinematics and muscle and (b) the prediction of survivability, are discussed in two articles in this Special Issue. In Contribution 9, Olikkal et al. (USA) fused kinematic and muscle synergies to improve motion reconstruction. They used a weighted linear combination of musculoskeletal synergies to reconstruct recorded kinematics and recorded muscle activities. They also evaluated results obtained using principal component analysis (PCA) and independent component analysis (ICA) to reduce dimensionality and found that ICA performed better for the analysed task. In Contribution 10, Sedighi-Maman et al. (USA) proposed an interpretable two-stage modelling approach for lung cancer survival prediction. A generalised linear model (GLM) is introduced as an interpretable block in their work, which can assist clinicians in decision making by prioritising the most salient factors related to prediction. Interpretability has attracted widespread attention in biomedicine in recent years as it can seriously limit the possibilities for practical adoption [12]. Therefore, in Contribution 6, the model’s interpretability is also emphasised by the attention mechanism.

## 7. Conclusions

In recent decades, artificial intelligence, especially machine learning, has made promising achievements in biomedicine. However, the use of appropriate strategies is important in the application of machine learning to biomedical data. This Special Issue collects ten research papers that provide general approaches to some biomedical problems and clear practical examples in different application scenarios. This collection of papers provides a good reference for the current state of the art and an excellent starting point for developing new advanced methods in the future.

## Figures and Tables

**Table 1:** List of the accepted papers.

No. of Contribution	Title	Focus	Type of Organization	Country
1	Prediction of Diabetes Mellitus Progression Using Supervised Machine Learning	Biomedical diagnostics	University	USA
2	Prediction of Preterm Labor from the Electrohysterogram Signals Based on Different Gestational Weeks	Biomedical diagnostics	University	Poland, Finlan, Lithuania
3	Spatiotemporal eye-tracking feature set for improved recognition of dyslexic reading patterns in children.	Biomedical diagnostics	University	Serbia
4	A machine learning approach for predicting non-suicidal self-injury in young adults	Biomedical diagnostics	University, Industry	Spain
5	Treatment Outcome Prediction Using Multi-Task Learning: Application to Botulinum Toxin in Gait Rehabilitation	Biomedical care tools	University, Laboratory	France, Pakistan
6	Enabling timely medical intervention by exploring health-related multivariate time series with a hybrid attentive model	Biomedical care tools	University,	China
7	Estimation of Respiratory Rate during Biking with a Single Sensor Functional Near-Infrared Spectroscopy (fNIRS) System	Health monitoring	University, Industry	Netherlands, Lithuania
8	HUMANISE: human-inspired smart management, towards a healthy and safe industrial collaborative robotics	Industrial applications	University, Laboratory	UK, Spain
9	Data Fusion-Based Musculoskeletal Synergies in the Grasping Hand	Others	University	USA
10	An Interpretable Two-Phase Modeling Approach for Lung Cancer Survivability Prediction	Others	University	USA
